# Dysregulated Autophagy Leads to Oxidative Stress and Aberrant Expression of ABC Transporters in Women with Early Miscarriage

**DOI:** 10.3390/antiox10111742

**Published:** 2021-10-30

**Authors:** Saira Shahnawaz, Usman Shah Nawaz, Jonas Zaugg, Ghulam Hussain, Nadia Malik, Muhammad Zahoor-ul-Hassan Dogar, Shoaib Ahmad Malik, Christiane Albrecht

**Affiliations:** 1 Department of Biochemistry, Sargodha Medical College, University of Sargodha, Sargodha 40100, Pakistan; sairanawaz14@gmail.com (S.S.); usman.shahnawaz@uos.edu.pk (U.S.N.); postdoc233@yahoo.com (M.Z.-u.-H.D.); 2 Institute of Biochemistry and Molecular Medicine, University of Bern, 3012 Bern, Switzerland; jonas.zaugg@ibmm.unibe.ch; 3 Department of Allied Health Sciences, Sargodha Medical College, University of Sargodha, Sargodha 40100, Pakistan; 4 Swiss National Centre of Competence in Research (NCCR) TransCure, University of Bern, 3012 Bern, Switzerland; 5 Neurochemicalbiology and Genetics Laboratory (NGL), Department of Physiology, Government College University, Faisalabad 38000, Pakistan; ghulamhussain@gcuf.edu.pk; 6 Department of Gynaecology and Obstetrics, Maula Bakhsh Teaching Hospital Sargodha, Sargodha 40100, Pakistan; drksn1@hotmail.com

**Keywords:** ATP-binding cassette (ABC) transporters, autophagy, antioxidant, early miscarriage, oxidative stress, placenta

## Abstract

Early miscarriage (EMC) is a devastating obstetrical complication. ATP-binding cassette (ABC) transporters mediate cholesterol transfer across the placenta and enhance cell survival by effluxing substrates from target cells in the presence of stressors. Recent evidence reports an intricate interplay between autophagy and ABC transporters. We hypothesized that dysregulated autophagy and oxidative stress (OS) in the placenta leads to abnormal expression of membrane transporters contributing to poor pregnancy survival in EMC. We determined mRNA and protein expression of autophagy genes (Beclin-1/Bcl-2/LC3I/LC3II/p62) and ABC transporters (ABCA1/ABCG1/ABCG2) in placentae from EMC patients (*n* = 20), term controls (*n* = 19), first trimester (*n* = 6), and term controls (*n* = 5) controls. Oxidative/antioxidant status and biomarkers of oxidative damage were evaluated in maternal serum and placentae from EMC and healthy controls. In EMC, placental expression of LC3II/LC3I as well as of the key autophagy regulatory proteins Beclin-1 and Bcl-2 were reduced, whereas p62 was increased. Both in the serum and placentae of EMC patients, total OS was elevated reflected by increased oxidative damage markers (8-OHdG/malondialdehyde/carbonyl formation) accompanied by diminished levels of total antioxidant status, catalase, and total glutathione. Furthermore, we found reduced ABCG1 and increased ABCG2 expression. These findings suggest that a decreased autophagy status triggers Bcl-2-dependent OS leading to macromolecule damage in EMC placentae. The decreased expression of ABCG1 contributes to reduced cholesterol export to the growing fetus. Increasing ABCG2 expression could represent a protective feedback mechanism under inhibited autophagy conditions. In conclusion, dysregulated autophagy combined with increased oxidative toxicity and aberrant expression of placental ABC transporters affects materno-fetal health in EMC.

## 1. Introduction

Early miscarriage (EMC), defined as the pregnancy loss during the first trimester (12 weeks), is one of the devastating obstetrical complications, which affects 10–15% of all clinically recognized pregnancies globally. About 80% of all clinically apparent miscarriages occur during the first trimester [1,2]. Around 50–60% of the early miscarriages are due to fetal chromosomal abnormalities, mainly trisomies [3]. Maternal diseases such as diabetes, acquired thrombophilia, and immune disorders, as well as lifestyle factors are associated with early pregnancy loss. However, approximately 40% of early miscarriages are classified as idiopathic, and the pathophysiology of these human early miscarriages has not been elucidated [4].

Autophagy represents a pathway that maintains intracellular protein quality controls and homeostasis. It constitutes a major protective mechanism allowing the cells to survive in response to various stressors, including nutritional or starvation stress, hypoxia, oxidative stress, endoplasmic reticulum (ER) stress, mitochondrial or DNA damage, and others [5,6]. Autophagy plays a vital role in early embryo and fetal development. Several studies report that autophagy is the crucial for healthy pregnancy from pre-implantation to placental development and embryo survival [7,8]. Research on placental autophagy suggests that an imbalance between its protective and destructive mechanism resulting in excessive or inhibited autophagy processes appears to be associated with pregnancy-related disorders including miscarriages [7,9]. Beclin-1 plays a central role in coordinating the cytoprotective function of autophagy through its interaction with class III phosphatidyl-inositol-3-phosphate kinase (PI3kIII/ Vps34) and participates in the early stages of autophagy, promoting the nucleation of the autophagic vesicle [10]. It is regulated by B-cell lymphoma/leukemia-2 (Bcl-2) that inhibits autophagy by binding and sequestering Beclin-1 under nutrient-rich conditions [6]. The execution of autophagy involves a group of proteins, known as autophagy-related proteins (ATG). Among these proteins, the yeast ATG-8 mammalian homologue microtubule-associated protein 1 light chain 3 (LC3) is essential for final autophagosome formation [11]. LC3 exists in two forms: a cytosolic form (LC3-I) and a lipid phosphatidylethanolamine-conjugated form (LC3-II) that is inserted into both the inner and outer membranes of the growing autophagosome [11,12]. Another widely used marker of autophagy is p62/sequestosome 1, which serves as an adaptor molecule implicated in the targeting of cargo for autophagosomes and is itself degraded by autophagy [12,13].

Considerable evidence has associated EMC with widespread trophoblastic oxidative stress (OS) as a common pathway leading to degeneration of the placenta, which is linked to the onset of defective and fluctuated premature maternal circulation all over the placenta affecting both fetus and mother [14,15]. Some studies ascertain that the aberrant levels of OS markers as well as increased apoptosis with decreased proliferation of trophoblastic tissue are associated with ECM [15,16]. Hence, in EMC, a substantial increase in the markers of OS, such as lipid peroxides, protein carbonyls and DNA damage, and diminished status of antioxidants (glutathione, vitamin C) along with compromised total antioxidant capacity (TAC) was detected [17,18,19]. Autophagy turned out to be a major regulator of OS in the placenta [9]. It has been speculated that autophagy might play a pro-survival role in trophoblastic tissue, as increased Beclin-1 expression and LC3 activation during the first trimester of pregnancy has been reported [20]. Interestingly, resveratrol, a potent autophagy inducer, increases LC3 and Beclin-1 levels and reduces apoptotic cells, thereby ensuring the normal biological functions of placental trophoblasts [21]. The proper interactions between autophagy and OS play an important role in placental homeostasis [9,15].

ATP-binding cassette (ABC) transporters are important for the regulation of lipid transport and homeostasis in various aspects [22,23]. Two important members of the ABC transporter family, ABCA1 and ABCG1, are essential transporters of cholesterol and toxic oxidation products of cholesterol (oxysterols) as well as of sphingolipids and phospholipids and are potentially involved in maternal–fetal cholesterol transport across the placenta [24,25,26]. During early gestation, maternal cholesterol is important for normal development such as for the formation of cell membranes, embryonic growth, fetal survival, and steroid hormone synthesis [27,28], which is crucial for fetal development and a healthy pregnancy [29]. Differential localization of ABCA1 and ABCG1 in villous trophoblasts determines the direction of cholesterol transport [30]. Another member of subfamily G of ABC transporters, ABCG2, is also highly expressed in the human placenta [31]. It has been shown to efflux a wide variety of drugs and chemotherapeutic agents and may play additional roles in cell survival on stress conditions [32]. Studies in trophoblast cells, animal models, and placental tissues revealed that ABCA1 and ABCG1 are relevant for placental and fetal development, and several common pregnancy disorders are associated with altered expression and activity of ABC transporters [30,33,34]. Although the commonly reported role for ABC transporters is their ability to enhance cell survival by effluxing substrates from target cells, recent studies suggest that certain transporters can also protect cells against apoptosis in the presence of stressors that are not substrates for the transporters [33,34]. Moreover, recent literature reported that some ABC transporters are annotated as lysosomal membrane proteins (LMPs) signifying a relationship with the intracellular endocytic pathway designated for recycling or lysosomal degradation [35]. In the same line, a novel role for ABCG2 beyond the conventional drug-efflux function has been suggested, since its expression enhanced stress-induced autophagy and cell survival in multiple tumor cell lines [32,34].

Considering the essential role of autophagy in early placental development and regulation of OS and the importance of cholesterol transporters in maintaining the successful pregnancy, we hypothesized that dysregulated autophagy and oxidative stress leads to abnormal expression of selected placental membrane transporters (ABCA1, ABCG1, and ABCG2) contributing to poor pregnancy survival in EMC. 

## 2. Materials and Methods

### 2.1. Chemicals and Reagents

o-Dianisidine dihydrochloride, ferrous ammonium sulfate, xylene orange, 2,4-di-nitrophenylhydrazine, 1,1,3,3 Tetraethoxypropane, ABTS^TM^, NADPH, glutathione reductase (GR), and Trolox (6-hydroxy-2,5,7,8-tetramethylchroman-2-carboxylic acid) were purchased from Sigma-Aldrich, Darmstadt, Germany. Trichloroacetic acid (TCA) and 5,5′-Dithiobis (2-nitrobenzoic acid) were obtained from Merck, Darmstadt, Germany. Thiobarbituric acid (TBA) was purchased from VWR, Dietikon, Switzerland. All other chemicals were of analytical grade. Pierce Protease inhibitor cocktail (A32953), Trizol reagent, RNAlater, and bicinchoninic acid assay (BCA assay) reagents were obtained from Thermo Fisher Scientific, Rockford, IL, USA. Lipid determination kits (Innoline Merck TCH, Innoline Merck Triglycerides, Darmstadt, Germany and Spinreact HDLc-D, St. Esteve de Bas, Girona, Spain), ELISA Kit for 8-OHdG (MyBioSource; MBS267161, San Diego, CA, USA), and antibodies used for immunoblotting are mentioned in the respective sections.

### 2.2. Study Groups and Sample Collection

In this study, 20 women with EMC (7–12 weeks, incomplete miscarriage), 19 women at full-term healthy pregnancy, and 20 women at 1st trimester healthy pregnancy (7–12 weeks of gestation, only serum collection) were enrolled at the Department of Gynecology and Obstetrics, Maula Bakhsh Teaching Hospital Sargodha, Pakistan. Moreover, for assessing a potential gestational-age-related effect, 6 women with 1st trimester elective termination of pregnancy and 6 term controls were recruited at the Department of Obstetrics and Gynecology, University Hospital Bern, Switzerland. Blood and placental tissue samples were obtained from term controls immediately after delivery and at the time of EMC and 1st trimester elective termination of pregnancy after obtaining the women’s written informed consent and approval of the University Hospital Research Ethics Committee (approval number Pakistan: SU/ORIC/57; approval number Switzerland: 178/03). Anthropometric characteristics and relevant clinical data were also collected. Exclusion criteria in this study were smoking, maternal diabetes, hypertension, polycystic ovary syndrome, pregnancy with fibroid, and hypothyroidism/ hyperthyroidism. Only placentae and blood samples from non-medicated, non-smoking women and women without pathologies were recruited in this study.

Maternal venous blood samples (10 mL) were taken from term controls, EMC, and 1st trimester into chilled tubes without EDTA for all studied biochemical assays. Serum was immediately obtained by whole blood centrifugation at 4 °C: 3000× *g* for 20 min and frozen at −80 °C until time of analysis. Placental tissues were collected as previously described [36]. Immediately after term caesarean section, 1st trimester elective termination of pregnancy, and reported EMC, villous placental tissue samples were taken and intensively washed in Dulbecco’s phosphate buffered saline (DPBS, Gibco) to remove the blood contamination. Tissue samples were, then, immediately snap-frozen in liquid nitrogen and stored at −80 °C for protein expression analysis and biochemical analysis until further analysis. For RNA isolation, placental tissues were stored in RNAlater (Thermo Fisher Scientific, Rockford, IL, USA) at 4 °C for 24 h and, then, at −80 °C. First trimester control serum samples were collected from Pakistan, and placental tissues from 1st trimester elective termination were taken from Switzerland due to religious limitations in Pakistan.

### 2.3. Determination of Maternal Lipid Levels

Lipid determination was performed in maternal serum from EMC, 1st trimester, and term controls in the clinical laboratory of Maula Bakhsh Teaching Hospital Sargodha, Pakistan. TCh, triglycerides, and HDL were determined via standard direct enzymatic–colorimetric assays in a semi-automatic analyzer BTS-350 (Innoline Merck TCH, Darmstadt, Germany; Catalogue No: 5175040001), (Innoline Merck Triglycerides, Darmstadt, Germany; Catalogue No: 5175140001), and (Spinreact HDLc-D, St. Esteve de Bas, Girona, Spain; Catalogue No: 1001096). LDL and VLDL cholesterol were calculated from TCh, triglyceride, and HDL levels by applying the Friedewald’s equation [37,38].

### 2.4. RNA Isolation, Reverse Transcription, and Quantitative RT-PCR Analysis

RNA isolation was performed in all placental tissues with a slight modification using a Trizol reagent kit (Invitrogen, Waltham, Massachusetts, USA) [39]. Approx. 50 mg placental tissues were preserved in 1 mL RNAlater until analysis. Before RNA extraction, placental tissues were quickly washed with Dulbecco’s phosphate buffered saline (DPBS, Gibco) to remove the RNAlater. Placental tissues were homogenized in cold Trizol reagent (1 mL) on ice with a TissueLyser LT (QIAGEN, Hilden, Germany) followed by phenol/chloroform extraction with centrifugation at 12,000× *g* for 15 min at 4 °C. RNA precipitation was performed by isopropanol (100%) with final washing by 75% ethanol (1 mL) followed by centrifugation at 7500× *g* for 5 min at 4 °C. After RNA pellet resuspension in RNase-free water, the total RNA concentration was measured using NanoDrop 1000 (Thermo Scientific, Waltham, Massachusetts, USA). All RNA samples included in the study had an OD 260/280 ratio > 1.8. The assessment of RNA integrity (qualitatively) was completed by using Agilent 2100 Bioanalyzer (Agilent Technologies, Santa Clara, CA, USA). Total RNA degradation was expressed as RNA integration numbers (RIN). Electrophoresis data were analyzed using the 2100 Expert Agilent software (Agilent Technologies, Santa Clara, CA, USA).

First-strand cDNA synthesis was performed from 2 μg of total RNA by using GoScript™ Reverse Transcriptase System (Promega, Dübendorf, Switzerland), according to the kit manufacturer’s instructions. Obtained cDNA was amplified by quantitative RT-PCR (RT-qPCR) reactions in 10 μL volume with 1 μL cDNA template and 0.5 μmol L^−1^ specific primers (primer nucleotide sequences are listed in Table S1 and 2× GoTaq^®^ PCR Master Mix (Promega). An amplification reaction was performed in duplicates on 384-well plates (Applied Biosystem) on the ViiA™ 7 Real-Time PCR System (Applied Biosystems, Waltham, MA, USA).

### 2.5. Assessment of Autophagy and ABC Transporters by Immunoblotting

Protein expression changes in the autophagy pathway (Beclin-1, Bcl-2, LC3-II, and p62,) and alterations in ABCG1 and ABCG2 on protein level were determined by immunoblotting. Placental tissues were homogenized in ice-cold hypotonic buffer (10 mmol L^−1^ Tris-HCl; 10 mmol L^−1^ NaCl; 1.5 mmol L^−1^ MgCl_2_; 0.1% TritonX-100; protease inhibitor cocktail (Sigma, Darmstadt, Germany); pH 7.4). The samples were centrifuged 1000× *g* for 10 min at 4 °C, and supernatants were collected in fresh tubes and stored upon at −80 °C until analysis. The protein content of tissue lysates was measured by Pierce bicinchoninic acid assay kit (BCA) using bovine serum albumin (BSA) as standard. 

Forty micrograms of the total tissue lysates were mixed with Laemmli sample buffer and loaded on 10% acrylamide gels for Beclin-1, Bcl-2, p62, ABCG1, and ABCG2. Twelve percent acrylamide gels were used for LC3-I and LC3-II and separated by SDS-PAGE. The immobilized bands were, then, semi-dry transferred to nitrocellulose membranes (GE Healthcare). Blots were blocked with 5% *w*/*v* nonfat milk in Tris Buffered Saline with 0.1% Tween-20 (TBST). The following primary antibodies were used: Beclin-1 rabbit mAB (Cell Signaling Technology 3495, Danvers, MA, USA, Bcl-2 mouse mAB (Cell Signaling Technology 15071, Danvers, Massachusetts, USA), Anti-LC3-I/II Antibody (Millipore ABC929, Darmstadt, Germany), SQSTM1/p62 Antibody (Cell Signaling Technology 5114S, Danvers, Massachusetts, USA), Anti-ABCG1 antibody (Genetex GTX81867, Irvine, CA, USA), Anti-ABCG2 antibody (Genetex GTX100437, Irvine, CA, USA), and mouse anti-β-actin antibody (Sigma A2228, Darmstadt, Germany) for the reference signal (loading control). Primary antibodies were incubated at 4 °C overnight by shaking followed by washing 4 times with TBST (Tris-buffered saline supplemented with 0.1% Tween 20) and, then, incubation with DyLight 680 or 800 fluorescence conjugated secondary antibodies (Thermo Fisher Scientific, Rockford, IL, USA). The immunoreactive specific bands for Beclin-1, Bcl-2, LC3-I, LC3-II, p62, ABCG1, and ABCG2 were densitometrically analyzed (Odyssey^®^ Sa Infrared Imaging System; LI-COR, Bad Homburg, Germany) and related to β-actin as internal control.

### 2.6. Placental Tissue Homogenates for Biochemical Assays

Approximately 300 mg placental tissue was taken with 1.5 mL ice-cold tissue extraction buffer (0.8% NaCl; 0.01 mol L^−1^ sucrose; 0.01 mol L^−1^ Tris-HCl; 0.0001 mol L^−1^ EDTA-2Na; protease inhibitor cocktail (Pierce, Rockford, IL, USA ); pH 7.4) followed by centrifugation for 10 min at 1000× *g* and 4 °C. The supernatant was collected in fresh tubes and stored upon analysis at −80 °C.

### 2.7. Total Oxidative Stress Assay (TOS)

TOS was measured in the serum samples and tissue homogenates from EMC, 1st trimester, and term controls by colorimetric method using o-dianisidine dihydrochloride as substrate described in the method [40]. Briefly, 50 μL of sample was added in R1 (Reagent 1: 150 μmol L^−1^ xylenol orange; 140 mmol L^−1^ NaCl; 1.35 mol L^−1^ glycerol; 25 mmol L^−1^ H_2_SO_4_ solution; pH 1.75) followed by the addition of R2 (Reagent 2: 5 mmol L^−1^ ferrous ammonium sulfate; 10 mmol L^−1^ o-dianisidine dihydrochloride; 25 mmol L^−1^ H_2_SO_4_ solution). The absorbance was taken by an automated analyzer (Biolab-310, Biorays, Faisalabad, Pakistan) at the wavelength 560 nm on the basis of the oxidation of ferrous ion to ferric ion in the presence of several oxidant species. The assay was calibrated with hydrogen peroxide.

### 2.8. Total Antioxidant Capacity Assay (TAC)

TAC was evaluated in serum samples and tissue homogenates from EMC, 1st trimester, and term controls by using a novel automated colorimetric method as described [41]. The more stable, colored 2,2′ -azinobis-3-ethylbenzothiazoline-6-sulfonic acid radical cation (ABTS^*+^) was used as substrate. Fifty microliters of sample were added in R1 (Reagent 1: 0.4 mol L^−1^ acetate buffer; pH 5.8) followed by the addition of R2 (Reagent 2: 10 mmol L^−1^ ABTS^*+^ in 30 mmol L^−1^ acetate buffer: pH 3.6). Antioxidants present in the sample accelerated the bleaching rate (color disappearance of ABTS^*+^) to a degree proportional to their concentrations. This reaction was recorded by an automated analyzer spectrophotometrically (Biolab-310) at a wavelength of 630 nm. The bleaching rate (color disappearance) was inversely related to the TAC of the sample. The assay was calibrated with Trolox (6-hydroxy-2,5,7,8-tetramethylchroman-2-carboxylic acid, see Section 2.1. Chemicals and Reagents). 

### 2.9. Enzymatic Antioxidant Assay by Measuring Catalase Activity

Enzymatic antioxidant potential was assessed by measuring the catalase specific activity in maternal serum and placental tissues, according to a described method [42]. Sample (serum and tissue homogenate) was added in a cuvette containing 50 mmol L^−1^ phosphate buffer (pH 7.0) and 30 mmol L^−1^ hydrogen peroxide (H_2_O_2_). Catalase activity was measured at 240 nm using a spectrophotometer for 1 min. The decomposition of H_2_O_2_ was directly followed by monitoring the decrease in absorbance. The catalase activity was determined by using the molar absorption coefficient of 43.6 mol L^−1^ cm. One unit of catalase activity is equal to 1 mmol H_2_O_2_ degraded per minute. Catalase-specific activity was expressed as U min^−1^ mg^−1^ protein.

### 2.10. Measurement of Total Glutathione Levels

The assay to estimate the antioxidant potency by measuring total GSH levels was performed, according to the method from Rahman et al 2007 [43]. Maternal serum and placental tissues were prepared as follows: Approximately 50 mg tissue was taken, and 1 mL extraction buffer (0.1 mol L^−1^ potassium phosphate buffer with 5 mmol L^−1^ EDTA disodium salt, 0.6% sulfosalicylic acid, 0.1% Triton X-100; pH 7.5) was added followed by centrifugation at 8000× *g* for 10 min at 4 °C. Supernatant was transferred in a new tube and used for the total GSH measurement. Fifty microliters serum was added in one-half volume of 0.6% sulfosalicylic acid followed by centrifugation at 4 °C and 8000× *g* for 10 min. The assay was based on the reaction of GSH with 5,5′-dithio-bis-2-nitrobenzoic acid (DTNB) that produces the chromophore 5-thio-2-nitrobenzoic acid (TNB) and oxidized glutathione–TNB adduct (GS–TNB). The rate of TNB formation was measured at 405 nm using Vmax® Kinetic ELISA Microplate Reader (Molecular Devices, VWR, Dietikon, Switzerland) and was proportional to the concentration of GSH in the sample. The disulfide product (GS–TNB) was, then, reduced by glutathione reductase (GR) in the presence of NADPH recycling GSH back into the reaction. The determination of total GSH concentration detects both forms of GSH, the reduced sulfhydryl form (GSH) and the oxidized glutathione disulfide (GSSG). The total GSH concentration was calculated from the linear standard curve based on a 1:2 dilution series ranging from 26.4 to 0.1 nmol ml^−1^. GSH contents (in placental tissues) were normalized to the protein concentration.

### 2.11. Measurement of the DNA Oxidative Stress Marker 8-OHdG

The ubiquitous marker of oxidative DNA damage, 8-OHdG, was measured by double-sandwich ELISA technique (MyBioSource; MBS267161, San Diego, CA, USA) in both serum and placental tissues from EMC, 1st trimester, and term control, according to the kit manufacturer’s instructions. Briefly, human 8-OHdG monoclonal antibody was pre-coated, and the detecting antibody was polyclonal and biotin labeled. Samples (serum, placental homogenates, and different concentrations of human 8-OHdG standard samples; 100 μL each for 90 min at 37 °C) and biotin-labeled antibody were added into ELISA plate wells followed by DPBS washing (60 min at 37 °C). Then, avidin–peroxidase conjugates were added to the ELISA wells (30 min, 37 °C). After washing, the ELISA plate with DPBS, TMB substrate (3,3′,5,5′-Tetramethylbenzidine) was added for coloring (30 min, 37 °C). TMB turns blue in a peroxidase catalytic reaction and, finally, turns yellow under the action of acid. The color depth and the factors to be quantified in the samples are positively correlated. The optical density (OD 450 nm) values were measured within 10 min by using Vmax® Kinetic ELISA Microplate Reader (olecular Devices, VWR, Dietikon, Switzerland). The 8-OHdG concentration of samples was determined using a standard curve based on a 1:2 dilution series ranging from 10 to 0.156 ng mL^−1^.

### 2.12. Measurement of Lipid Peroxidation Products (TBARS)

Lipid peroxidation products, such as MDA, were measured by using a thiobarbituric acid reactive substances (TBARS) assay in maternal serum and placental tissue homogenates. MDA combines with thiobarbituric acid (TBA) in a 1:2 stoichiometry to form a fluorescent adduct. TBARS were expressed as MDA equivalents and normalized to total protein concentration (tissue homogenates) [44]. Concisely, 15% *w*/*v* trichloroacetic acid (TCA), sample (serum and tissue homogenate), or MDA standard (1,1,3,3 Tetraethoxypropane), 0.67% *w*/*v* TBA in 2.5 mol L^−1^ HCl were mixed in a 4:5:8 ratio followed by vortexing and boiled at 95 °C for 20 min. The MDA standard curve was prepared from 2 to 0.007 nmol mL^−1^ as a 1:2 dilution series. After cooling the samples to room temperature, 1-butanol (2 mL) was added followed by gentle mixing. For phase separation, samples were spun at 1000 g for 1 min. For the measurement, the butanol phase (top) was transferred to a 96-well black-wall plate and measured at an excitation/emission wavelength of 530/550 nm, respectively, by using Molecular Devices Flex Station II fluorescence microplate reader (VWR, Dietikon, Switzerland). MDA equivalents were calculated by interpolation to MDA standard curve.

### 2.13. Measurement of Protein Carbonylation

OS damage on the protein level was evaluated using a protein carbonylation assay. Carbonyl groups (aldehydes and ketones) are produced on protein side chains when they are oxidized. The detection of protein carbonyl groups involves their reaction with 2,4-di-nitrophenylhydrazine (DNP), which leads to the formation of a stable 2,4-dinitrophenylhydrazone product, followed by its spectrophotometric quantification [45]. Sample (serum and placental homogenate) was mixed with 2.5% *w*/*v* TCA and centrifuged for 3 min at 11,000× *g*. Five hundred microliters of 10 mmol L^−1^ DNP in 2 mol L^−1^ HCl was added to the precipitates and allowed to stand for 1 h at room temperature, with vortexing every 10 to 15 min. Thereafter, 500 μL of 20% *w*/*v* TCA was added for precipitation followed by washing (3 steps) with ethanol-ethylacetate (1:1) to remove extra reagent. Six hundred microliters of 6 mol L^−1^ guanidine solution was added to precipitated proteins to dissolve. The carbonyl content was determined by taking the spectra at OD_405nm_ by Vmax® Kinetic ELISA Microplate Reader (Molecular Devices, VWR, Dietikon, Switzerland) using a molar absorption coefficient of 22,000 mol L^−1^ cm^−1^. The protein carbonyl contents were expressed as nmol mg^−1^ protein normalized to the protein concentration. 

### 2.14. Statistical Analysis

Anthropometric, clinical data and the lipid profiles between EMC, 1st trimester controls, and term controls were evaluated by Kruskal–Wallis tests followed by Dunn’s multiple comparison. For the statistical analysis of mRNA levels to determine the autophagy genes and ABC transporters, two-tailed Mann–Whitney tests (1st trimester vs. term controls and EMC vs. term controls) were performed. Data were represented as Tukey box and whiskers, mean (x) and median (-). Placental protein levels to assess the autophagy regulatory proteins, autophagy markers, and ABCG1 and ABCG2 transporters were compared between 1st trimester vs. term control and EMC vs. term control by two-tailed Mann–Whitney tests. TOS, antioxidant potential levels (TAC and enzymatic antioxidant assay), GSH levels, and biochemical markers of DNA, lipid, and protein oxidative damage in maternal serum were compared between EMC, 1st trimester, and term controls by Kruskal–Wallis tests followed by Dunn’s multiple comparison. For statistical analysis of placental OS levels and antioxidant potential in placentae determined in EMC vs. term controls and 1st trimester vs. term control, two-tailed Mann–Whitney tests were applied, respectively. Asterisks in the figures indicate significance levels: ‡ = *p* ≤ 0.001, † = *p* ≤ 0.01, * = *p* ≤ 0.05. Statistical comparisons were performed using GraphPad Prism software (GraphPad Software, Inc. La Jolla, CA, USA).

## 3. Results

### 3.1. Maternal Lipid Profile and Clinical Parameters of EMC Patients

**Figure 1:**
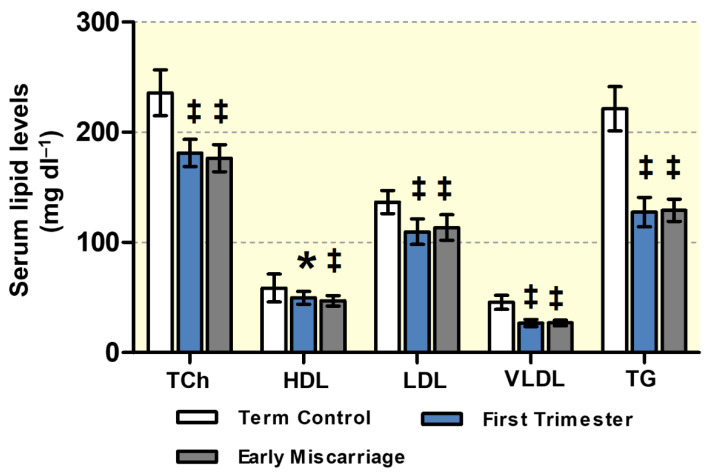
**Maternal serum lipid concentrations**. Total cholesterol (TCh), high-density lipoprotein (HDL), and triglycerides (TG) were determined in the serum of term controls, first trimester controls, and early miscarriage patients (*n* = 19, *n* = 20, and *n* = 20, respectively) by using standard direct enzymatic–colorimetric assays. Low-density lipoprotein (LDL) and very low-density lipoprotein (VLDL) cholesterol were calculated from Friedewald’s equation by using TCh, TG, and HDL levels. Data are presented as mean ± SD for each group. No statistically significant differences between first trimester and early miscarriage (EMC) were found. Statistical analysis was performed by using Kruskal–Wallis test followed by Dunn’s multiple comparison test; ‡ (*p* ≤ 0.001), * (*p* ≤ 0.05).

As reported in Table 1, no difference was found in the general maternal parameters between the EMC, first trimester, and term control groups. There were no significant differences in weight and BMI between the EMC and first trimester groups, although both groups presented with significantly reduced weight and BMI as compared to healthy term (third trimester) controls based on physiological weight gain during pregnancy. Similarly, maternal total cholesterol (TCh), triglyceride (TG), high-density lipoprotein (HDL), low-density lipoprotein (LDL), and very low-density lipoprotein (VLDL) levels in both EMC and first trimester group were significantly lower as compared to the term control group. However, no differences were found between EMC patients and first trimester controls (Table 1, Figure 1).

### 3.2. mRNA and Protein Expression of Autophagy Markers in EMC

**Figure 2:**
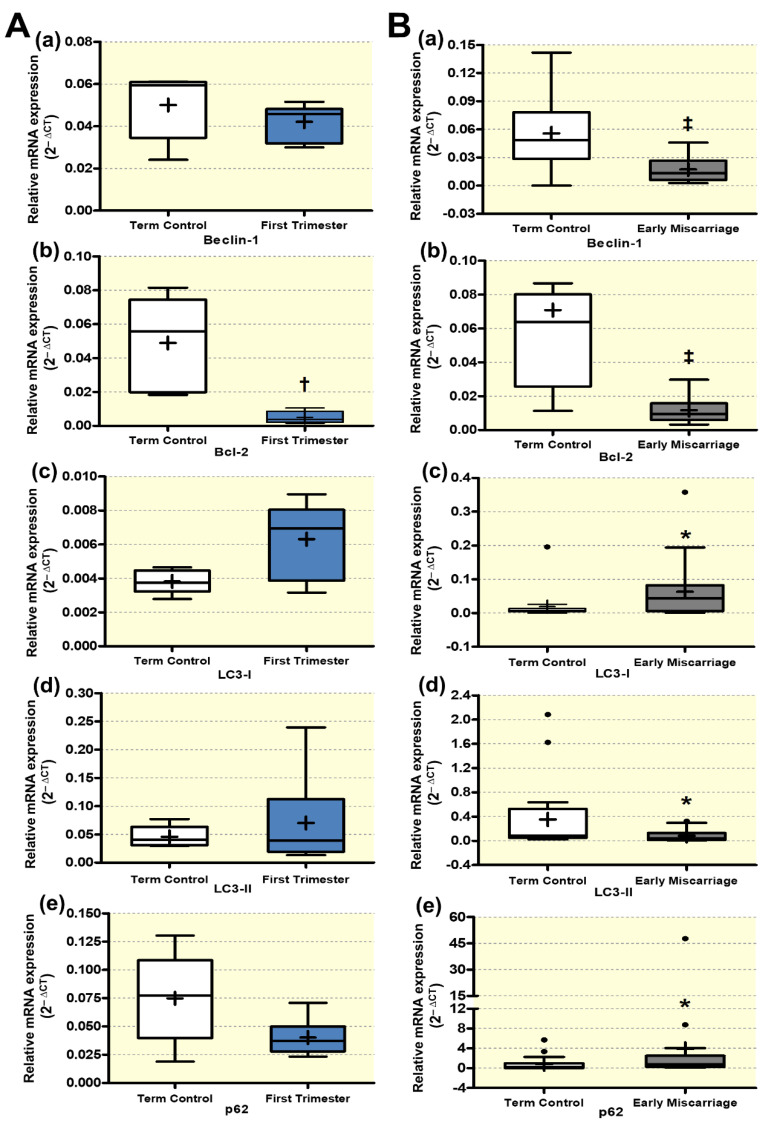
**qRT-PCR analysis of autophagy genes.** qRT-PCR of several well established autophagy genes (**a**–**e**) was performed in placentae from (**A**) first trimester and term controls (*n* = 6 and *n* = 5, respectively; Swiss cohort), (**B**) EMC patients and term controls (*n* = 20 and *n* = 19, respectively). mRNA expression determined by RT-qPCR is presented as 2^−ΔCT^ (ΔCT= CT value of target gene—CT value of the mean of β-actin, GAPDH, YWHAZ, B2MG, L19, hTBP, hsUBQ). Data are presented as mean (x), median (-), and Tukey box and whiskers (1.5 times interquartile range). Decreased autophagy status was detected in EMC patients. Statistical analysis was performed by using two-tailed Mann–Whitney test for comparing first trimester vs. term controls (**A**) and EMC vs. term controls (**B**); ‡ (*p* ≤ 0.001), † (*p* ≤ 0.01), * (*p* ≤ 0.05). Abbreviations: *β-actin*, beta-actin; *GAPDH*, Glyceraldehyde-3-phosphate dehydrogenase; *YWHAZ*, 14-3-3 protein zeta/delta; *B2MG*, beta-2 microglobulin; *L19* (RPL19), ribosomal protein L19; *TBP*, TATA-box-binding protein; *UBC*, ubiquitin.

To assess the autophagy status in EMC patients, we analyzed the autophagy genes by qRT-PCR in placentae from EMC, first trimester controls, and term controls. Due to religious and ethical restrictions in Pakistan, the ECM placentae could only be compared to a healthy term control cohort (Figure 2B). To rule out potential gestational-age-related differences between the first trimester, when ECM samples were taken, and term controls, a separate set of first trimester and term controls collected in Switzerland were compared for gestational-age-dependent expression effects (Figure 2A). For the sake of clarity and since we cannot rule out ethnically based differences in the expression patterns, the two sets of samples from Pakistan and Switzerland are shown separately. Beclin-1, LC3-I, LC3-II, and p62 mRNA abundances were similar, while Bcl-2 downregulated in first trimester compared to term control placentae (Figure 2A, panels a–e). Beclin-1, Bcl-2, and LC3-II were significantly downregulated, while LC3-I and p62 were significantly increased on the transcriptomic level in EMC patients compared to term controls (Figure 2B, panels a–e).

**Figure 3:**
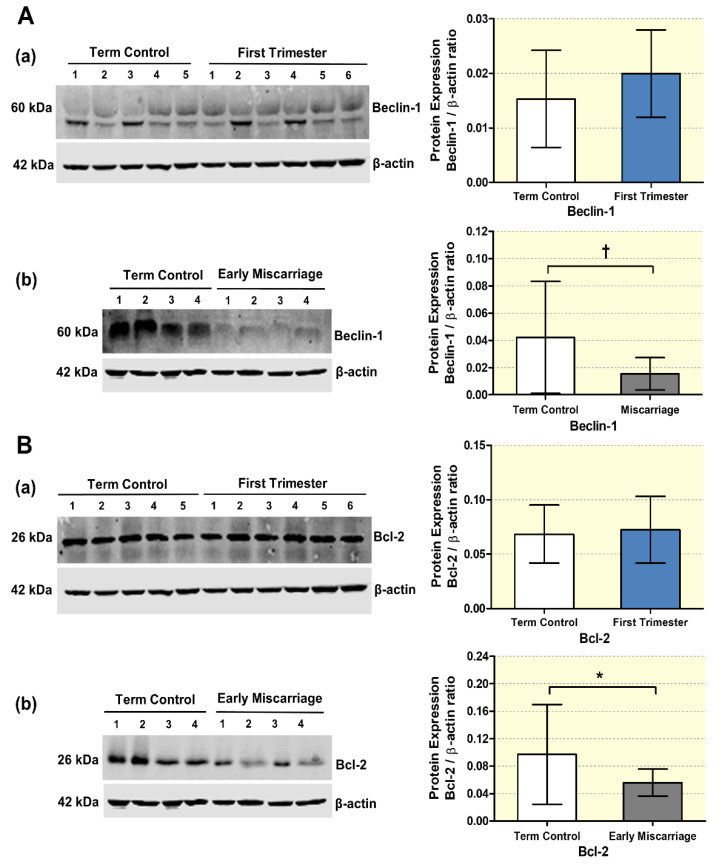
**Protein expression changes in autophagy regulatory proteins in placental tissues**. (**A**) Beclin-1, (**B**) Bcl-2 expression visualized by immunoblotting in placentae from (**a**) first trimester and term controls (*n* = 6 and *n* = 5, respectively; Swiss cohort), (**b**) EMC patients and term controls (*n* = 20 and *n* = 19, respectively) were quantified by fluorescence detection analysis and normalized to β-actin as loading control. Results of representative immunoblots for autophagy regulatory proteins are shown. Reduction in the autophagy regulatory proteins Beclin-1 and Bcl-2 was detected in EMC patients. Statistical analysis was performed by using two-tailed Mann–Whitney test for comparing first trimester vs. term controls (Swiss cohort) (**a**) and EMC vs. term controls (**b**); † (*p* ≤ 0.01), * (*p* ≤ 0.05).

**Figure 4:**
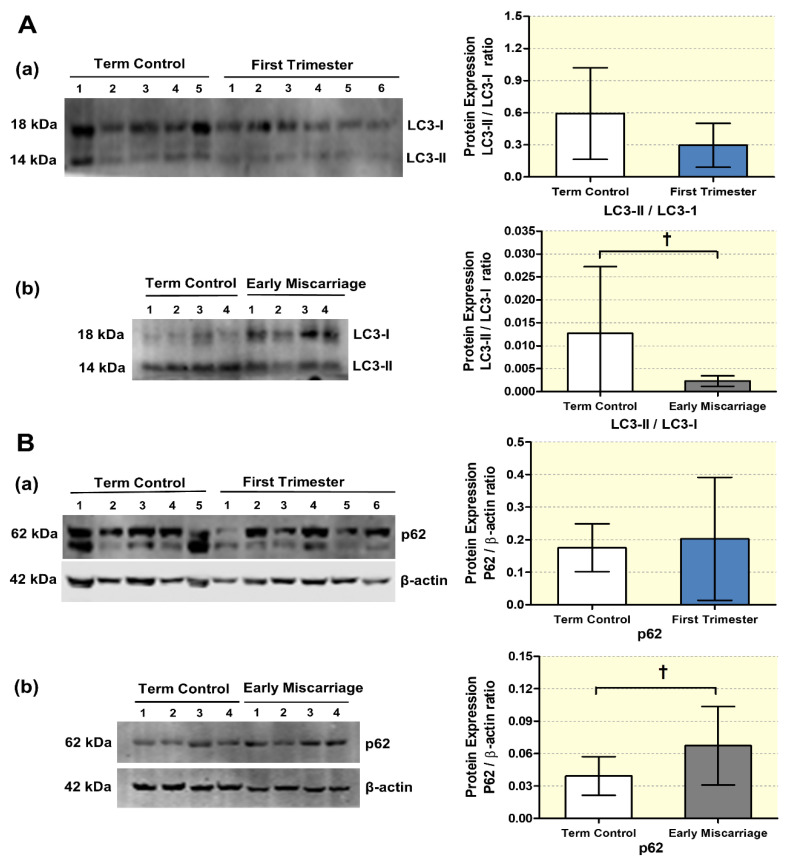
**Protein expression changes in autophagy makers in placental tissues**. (**A**) LC3-II, (**B**) p62 expression visualized by immunoblotting in placentae from (**a**) first trimester and term controls (*n* = 6 and *n* = 5, respectively; Swiss cohort), (**b**) EMC patients and term controls (*n* = 20 and *n* = 19, respectively) were quantified by fluorescence analysis and normalized to β-actin as loading control. Results of representative immunoblots for autophagy markers are shown. Reduction in the autophagy markers LC3II/LC3I and increased p62 were detected in EMC patients. Statistical analysis was performed by using two-tailed Mann–Whitney test for comparing first trimester vs. term controls (Swiss cohort) (**a**) and EMC vs. term controls (**b**); † (*p* ≤ 0.01).

Furthermore, to confirm the differential expression of autophagy regulatory proteins (Beclin-1, Bcl-2) and autophagy markers (LC3-II/LC3I, p62) also on the protein level, Western blot analysis was performed in EMC, first trimester, and term control placentae (Figure 3A,B and Figure 4A,B). No significant differences were detected between first trimester and term control placentae for Beclin-1, Bcl-2, LC3-II/LC3I, and p62 (Figure 3A(a),B(a) and Figure 4A(a),B(a)). In contrast, immunoblot analysis demonstrated downregulation of Beclin-1, Bcl-2, and the LC3II/LC31 ratio in EMC patients, while p62 protein expression was increased (Figure 3A(b),B(b) and Figure 4A(b),B(b)). Taken together, both the gene and protein expression patterns indicate reduced autophagy in EMC patients.

### 3.3. Altered Maternal Systemic and Placental Oxidative Stress

**Figure 5:**
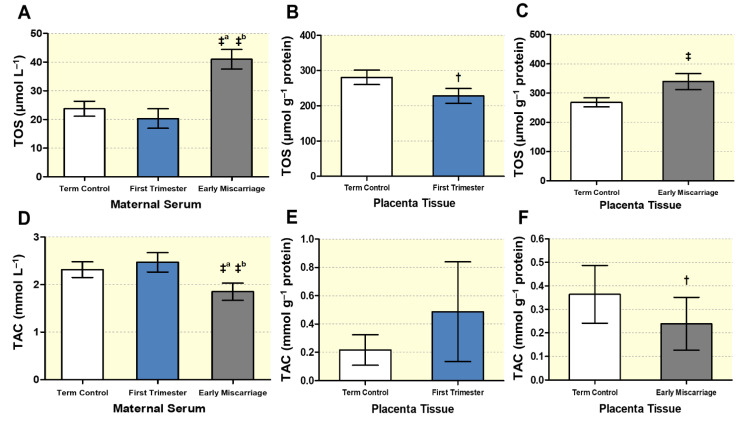
**Determination of total oxidative stress (TOS) and total antioxidant capacity (TAC).** TOS was determined in (**A**) maternal serum of first trimester, EMC patients, and term controls (*n* = 20, *n* = 20, and *n* = 19, respectively), (**B**) placentae of first trimester and term controls (n = 6 and n = 5, respectively; Swiss cohort), and (**C**) placentae of EMC and term controls (n = 20 and n = 19, respectively). Analysis of total antioxidant capacity (TAC) in (**D**) maternal serum of first trimester, EMC patients, and term control (*n* = 20, *n* = 20, and *n* = 19, respectively), (**E**) placentae of first trimester and term controls (*n* = 6 and *n* = 5, respectively; Swiss cohort), and (**F**) placentae of EMC and term control (*n* = 20 and *n* = 19, respectively). Data are shown as mean ± SD for each group. In (**A**,**D**), statistical analysis was performed by using Kruskal–Wallis test followed by Dunn’s multiple comparison test. In (**B**,**E**) and (**C**,**F**), statistical evaluation was performed by using two-tailed Mann–Whitney test for comparing first trimester vs. term controls (Swiss cohort) and EMC vs. term controls, respectively. ‡ (*p* ≤ 0.001), † (*p* ≤ 0.01). ‡^a^: significance between EMC and term control; ‡^b^: significance between EMC and 1st trimester.

The levels of total oxidative stress (TOS), TAC, and its related enzyme activity were measured in maternal serum and placentae of EMC, first trimester, and term controls. TOS was significantly higher in the EMC maternal serum (40.98 ± 3.41 μmol L^−1^) compared to first trimester (20.31 ± 3.39 μmol L^−1^) and term controls (23.71 ± 2.59 μmol L^−1^) (Figure 5A). Increased TOS levels were also detected in the EMC placentae compared to controls (339.7 ± 27.48, 280.6 ± 15.61 μmol g^−1^ protein, respectively) (Figure 5C). This increase was not due to a gestational-age effect, since the TOS level was significantly lower in first trimester placental tissues compared to term controls (227.7 ± 21.29, 280.6 ± 20.44 μmol g^−1^ protein, respectively) (Figure 5B). To investigate the antioxidant potential, we measured TAC in maternal serum and placentae of EMC compared to first trimester control and term control. There were significantly reduced TAC levels both in serum and placental tissues of EMC patients (1.85 ± 0.18 mmol L^−1^, 0.24 ± 0.11 mmol g^−1^ protein, respectively) (Figure 5D,F). These changes were not gestational-age-dependent, since no significant differences in TAC were found between first trimester and term controls in serum (2.47 ± 0.20 mmol L^−1^ vs. 2.32 ± 0.16 mmol L^−1^; Figure 5D) and placentae (0.49 ± 0.35 vs. 0.22 ± 0.1 mmol g^−1^ protein; Figure 5E).

**Figure 6:**
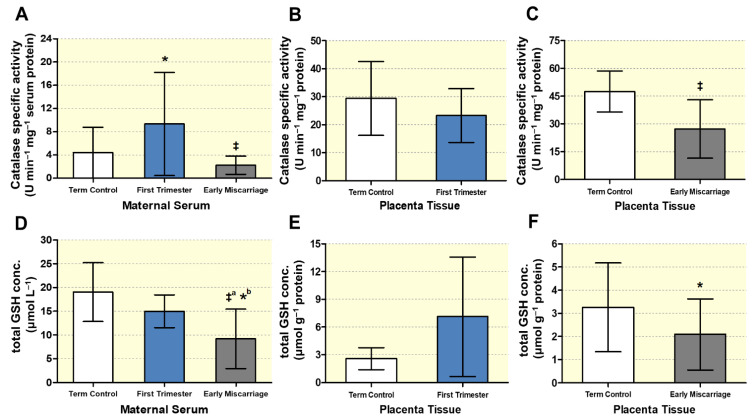
**Analysis of enzymatic antioxidant capacity**. Catalase activity was measured in (**A**) maternal serum of EMC patients (*n* = 20) and first trimester and term controls (*n* = 20, *n* = 19), placentae from (**B**) first trimester and term controls (*n* = 6 and *n* = 5, respectively; Swiss cohort), and (**C**) placentae from EMC patients and term controls (*n* = 20 and *n* = 19, respectively). Total glutathione (GSH) levels were determined in (**D**) maternal serum of EMC patients (*n* = 20) and first trimester and term controls (*n* = 20 and *n* = 19, respectively), (**E**) placentae of first trimester and term controls (*n* = 6 and n = 5, respectively; Swiss cohort), and (**F**) placentae of EMC and term control (*n* = 20 and *n* = 19, respectively). Data are shown as mean ± SD for each group. For (**A**,**D**), the statistical differences were analyzed using Kruskal–Wallis test followed by Dunn’s multiple comparison test. For (**B**,**E**) and (**C**,**F**), statistical analysis was performed by using unpaired two-tailed Mann–Whitney test for comparing first trimester vs. term controls (Swiss cohort) and EMC vs. term controls, respectively; ‡ (*p* ≤ 0.001), * (*p* ≤ 0.05), ‡: significance between EMC and first trimester; ‡^a^: significance between EMC and term control; *^b^: significance between EMC and 1st trimester.

Moreover, we studied the enzymatic antioxidant activity by analyzing catalase activity in EMC, first trimester, and term controls (Figure 6A–C). Maternal serum of EMC showed significantly decreased catalase activity (2.25 ± 1.57 U min^−1^ mg^−1^ protein), while the same was increased in first trimester serum (9.33 ± 8.87 U min^−1^ mg^−1^ protein) compared to term controls (4.41 ± 4.37 U min^−1^ mg^−1^ protein; Figure 6A). In the EMC placentae, catalase activity was significantly reduced compared to term controls (27.29 ± 15.74 vs. 47.50 ± 11.08 U min^−1^ mg^−1^ protein, respectively), while no differences were observed between first trimester placental tissues and term controls (Figure 6B,C).

As an additional indication for OS, total glutathione (GSH) levels were determined in maternal serum and placental tissues (Figure 6D–F). In EMC, total GSH levels were significantly reduced both in the serum (9.18 ± 6.24 vs. 14.97 ± 3.44 vs. 19.02 ± 6.19 μmol L^−1^ for ECM vs. first trimester vs. term control; Figure 6D) and in placentae (2.09 ± 1.53 vs. 3.26 ± 1.91 μmol g^−1^ protein, ECM vs. term control; Figure 6F). There was no significant difference in total GSH levels between first trimester (7.11 ± 6.45 μmol g^−1^ protein) and term control placentae (2.58 ± 1.18 μmol g^−1^ protein; Figure 6E).

### 3.4. Biochemical Markers of DNA, Lipid, and Protein Oxidative Damage

**Figure 7:**
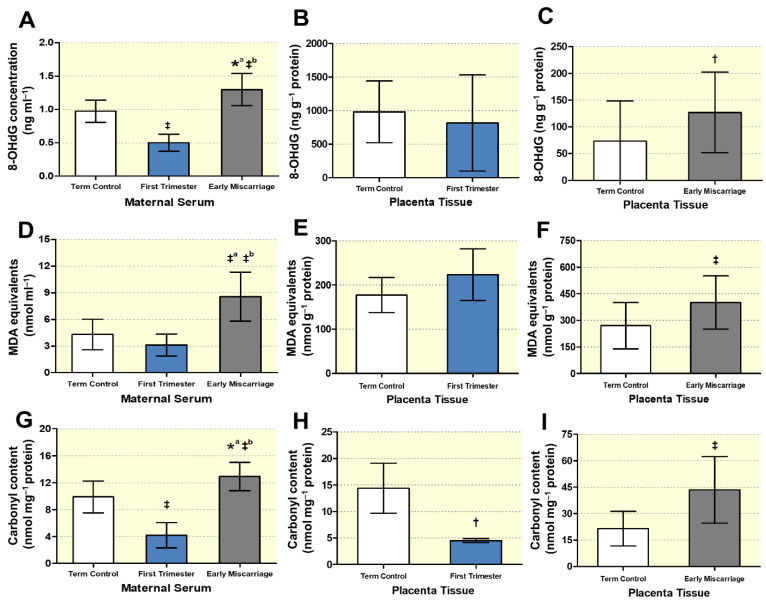
**Analysisof oxidative stress markers.** (**A**)–(**C**): Measurement of 8-OHdG protein levels by ELISA in (**A**) maternal serum of term controls, first trimester, and EMC (n = 19, n = 20, and n = 20, respectively), (**B**) placental tissue of first trimester and term controls (n = 5 and n = 6, respectively; Swiss cohort), and (**C**) placental tissue of EMC patients and term controls (*n* = 19 and *n* = 20, respectively). (**D**–**F**): Analysis of lipid oxidative damage as formation of malondialdehyde (MDA) equivalents in (**D**) maternal serum of 1st trimester, EMC patients, and term control (n = 20, n = 20, and n = 19, respectively), (**E**) placentae of first trimester and term controls (n = 6 and n = 5, respectively; Swiss cohort), and (**F**) placentae of EMC and term control (*n* = 20 and *n* = 19, respectively). (**G**–**I**): Measurement of protein carbonylation in (**G**) maternal serum of term controls, 1st trimester, and EMC (*n* = 19, *n* = 20, and *n* = 20, respectively), (**H**) placentae from first trimester and term controls (*n* = 6 and *n* = 5, respectively; Swiss cohort), and (**I**) EMC patients and term controls (*n* = 20 and *n* = 19, respectively). Data are presented as mean ± SD for each group. The statistical analysis in (**A**,**D**,**G**) was performed by Kruskal–Wallis test followed by Dunn’s multiple comparison test. In (**B**,**C**,**E**,**F**,**H**,**I**), statistical evaluation was performed by using the two-tailed Mann–Whitney test for comparing first trimester vs. term controls (Swiss cohort) and EMC vs. term controls, respectively. ‡ (*p* ≤ 0.001), † (*p* ≤ 0.01), * (*p* ≤ 0.05). ‡^a^: significance between EMC and term control; *^a^ significance between EMC and term control; ‡^b^: significance between EMC and first trimester.

We further analyzed the oxidative damage to biological macromolecules such as DNA, lipids, and proteins in EMC by measuring the DNA oxidative stress marker 8-OHdG, the production of malondialdehyde (MDA) equivalents as marker for lipid peroxidation, and carbonyl formation, respectively. As discussed before, since we cannot rule out ethnically based differences between the Swiss and the Pakistani population, the results for 8-OHdG, MDA, and carbonyl content are shown separately for the two cohorts. The levels of 8-OHdG were significantly higher in the maternal serum of EMC patients compared to both first trimester and term controls (Figure 7A). Interestingly, first trimester 8-OHdG serum concentrations were also significantly lower than in term controls (Figure 7A). Likewise, placental 8-OHdG was significantly higher in EMC, but there was no difference between first trimester and term controls (Figure 7B,C). Significantly increased lipid peroxidation (expressed as increased MDA formation) and protein carbonylation levels were detected both in maternal sera and placentae of EMC patients (Figure 7D, Figure 7F,G, Figure 7I, respectively). These increases cannot be attributed to a gestational age effect: lipid peroxidation in first trimester sera and placentae showed no difference compared to term controls (Figure 7D,E), and carbonyl content in first trimester serum and placental tissue was even lower than in term controls (Figure 7G,H).

### 3.5. Altered Expression of ABC Transporters

**Figure 8:**
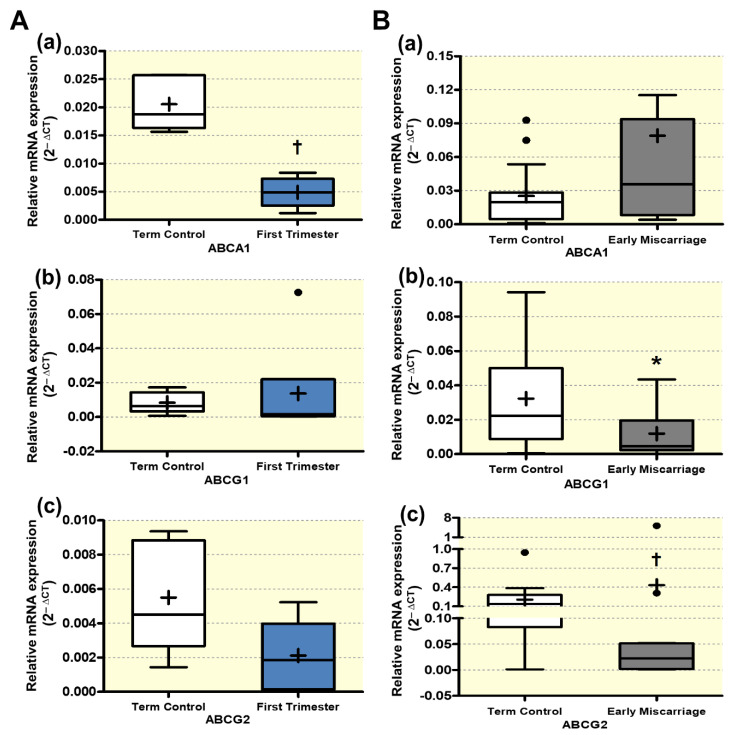
**qRT-PCR analysis of ABC transporters**. mRNA expression of selected ABC transporters was measured in placentae from (**A**) first trimester and term controls (*n* = 6 and *n* = 5, respectively; Swiss cohort) and (**B**) EMC patients and term controls (*n* = 20 and *n* = 19, respectively). mRNA abundances of (**a**) ABCA1, (**b**) ABCG1, and (**c**) ABCG2 are presented as 2^−ΔCT^ (ΔCT= CT value of target gene—CT value of the mean of reference genes β-actin, GAPDH, YWHAZ, B2MG, L19, hTBP, and hsUBQ). Data are presented as mean (x), median (-), and Tukey box and whiskers (1.5 times interquartile range). ABCG1 was significantly lower, whereas ABCG2 mRNA expression increased in ECM. Statistical significance was evaluated by using two-tailed Mann–Whitney test for comparing first trimester vs. term controls (Swiss cohort) (**A**) and EMC vs. term controls (**B**); † (*p* ≤ 0.01), * (*p* ≤ 0.05). Abbreviations: *β-actin*, beta-actin; *GAPDH*, Glyceraldehyde-3-phosphate dehydrogenase; *YWHAZ*, 14-3-3 protein zeta/delta; *B2MG*, beta-2 microglobulin; *L19* (RPL19), ribosomal protein L19; *TBP*, TATA-box-binding protein; *UBC*, ubiquitin.

We, then, explored the effect of impaired autophagy and increased OS on ABC transporters (ABCA1, ABCG1, and ABCG2) in EMC patients. Importantly, expression patterns of ABC transporters may vary between the Swiss and the Pakistani cohort due to genuine differences between the two ethnical populations. However, the Swiss first and term control groups only serve to detect potential expression differences related to the gestational age and not between different ethical populations. Therefore, the final interpretation of the results should not be majorly affected. The mRNA abundance of ABCG1 was significantly lower, whereas ABCG2 mRNA was increased in ECM (Figure 8B(b),(c), respectively). ABCA1 transcript levels showed a non-significant trend towards an increase in EMC compared to term controls (Figure 8B(a)). This difference is expected to be even more pronounced and reach significance if ABCA1 expression levels in ECM would be compared to ethnically matched first trimester controls. Indeed, in the Swiss control cohort, ABCA1 expression was significantly reduced in first trimester compared to term control placentae (Figure 8A(a), whereas no significant differences were found for ABCG1 and ABCG2 (Figure 8A(b),(c), respectively).

**Figure 9:**
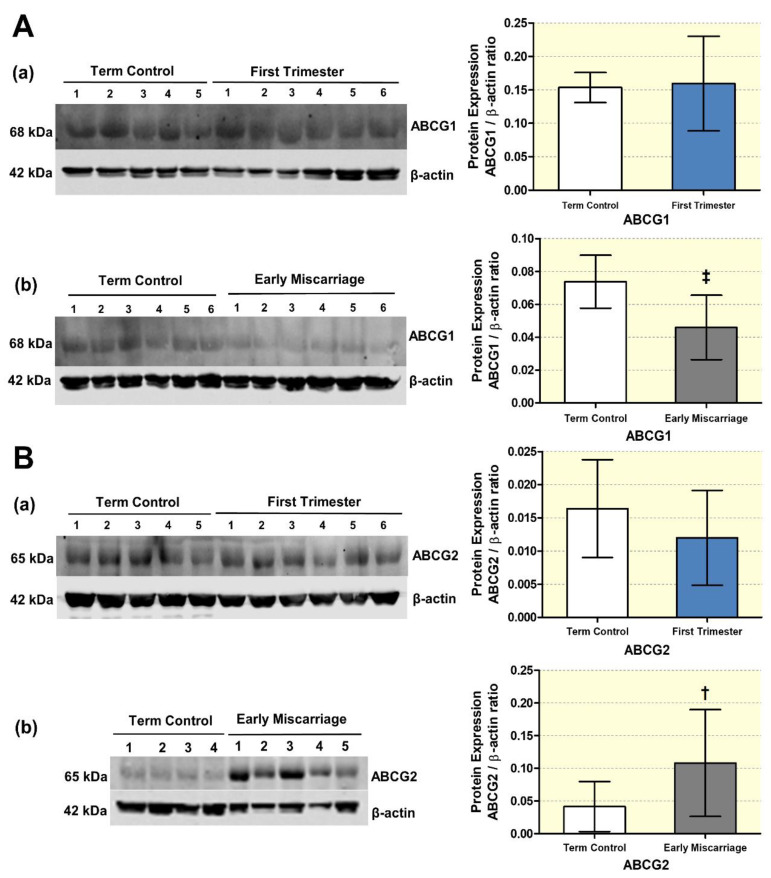
**Protein expression changes in ABC transporters in placental tissues.** (**A**) ABCG1 and (**B**) ABCG2 expression visualized by immunoblotting in placentae from (**a**) first trimester and term controls (*n* = 6 and *n* = 5, respectively, Swiss cohort) and (**b**) EMC patients and term controls (*n* = 20 and *n* = 19, respectively) was quantified by fluorescence detection analysis and normalized to β-actin as loading control. Results of representative immunoblots for ABCG1 and ABCG2 are shown. Reduced ABCG1 and increased ABCG2 protein levels were observed in EMC patients. Statistical analysis was performed by using two-tailed Mann–Whitney test for comparing first trimester vs. term controls (**a**) and EMC vs. term controls (**b**); ‡: *p* ≤ 0.001, †: *p* ≤ 0.01).

Those ABC transporters that we found significantly regulated on transcript level (ABCG1 and ABCG2) were further evaluated on protein level by performing Western blotting with consecutive densitometric analysis. β-actin served as loading control, and all signals were normalized to this protein. Immunoblotting revealed downregulation of ABCG1 and a clear induction of ABCG2 protein expression in placentae of EMC patients (Figure 9A(b) and Figure 9B(b), respectively). Similar to the mRNA results, no significant differences were detected between first trimester and term placenta neither for ABCG1 nor for ABCG2 (Figure 9A(a) and Figure 9B(a), respectively). These findings demonstrate that ABCG1 and ABCG2 showed comparable expression patterns on the transcript and protein level and are differentially regulated in EMC patients (Figure 8 and Figure 9).

## 4. Discussion

In this study, we investigated the role of autophagy in mediating OS and their involvement in the expression of ABC transporters (ABCA1, ABCG1, and ABCG2) in the placenta of pregnant women with EMC. We studied these mechanisms in an EMC and healthy term control cohort from Pakistan with a balanced distribution of maternal age, weight, and BMI. Due to ethical and religious restrictions, we were not able to collect gestational-age-matched first trimester placenta samples. To discriminate EMC-associated effects in the placenta from potential gestational-age-related changes, we separately studied a Swiss cohort of first trimester and term controls. We demonstrated aberrant autophagy and increased OS in EMC, which was accompanied by abnormal expression of placental ABC transporters. In this context, it is tempting to speculate that dysregulated autophagy processes in EMC enhance OS, which impacts the expression and function of ABC transporters. 

Autophagy is a homeostatic, recycling, and quality-control process of organelles and intracellular proteins by degrading the aged, damaged organelles and macromolecules [6]. It is proposed that autophagy is involved in certain cytosolic rearrangements that are required during embryogenesis and, thus, plays important roles in placentation, maintaining placental development, and normal pregnancy [46]. Since balanced levels of autophagy are critical for placentation, appropriate regulation of placental autophagy is crucial for normal placental function and fetal survival [12,47]. Studies have demonstrated the presence of autophagy in the placenta throughout gestation, but different levels have been reported in the first trimester and term normal human placenta [47,48]. We quantified levels of autophagy in EMC patients at both the transcript level and protein level by assessing the autophagy regulatory proteins Beclin-1 and Bcl-2 and the autophagy protein markers LC3II, LC3I, and p62. 

Our results indicate that, in EMC placentae, the autophagic pathway was inhibited as compared to gestational-age-matched first trimester controls and term controls. During the formation of the autophagosome and with the progression of autophagy, cytosolic microtubule-associated LC3I is cleaved and conjugated with phosphatidylethanolamine leading to the formation of LC3II, which is the autophagic vacuole-associated form. Importantly, decreased conversion of LC3I to LC3II or increased levels of LC3I are characteristic of reduced levels of autophagy. In contrast, higher levels of the LC3II protein are found when autophagy is increased, and an elevation in the LC3II/LC3I ratio has been considered a hallmark of autophagy. On the protein level, we detected a decreased LC3II/LC3I ratio in ECM. Our LC3II expression data, thus, suggest that autophagy levels are reduced in first trimester samples in comparison to normal-term placentae and are even further compromised in EMC patients. The protein p62, another widely used autophagy marker also known as sequestosome 1, showed increased expression levels in EMC placentae, suggesting the accumulation of p62 due to defects in the autophagy pathway. The observation of significantly diminished autophagy in placental villi of recurrent spontaneous abortions compared with healthy pregnancies suggests that autophagy inhibition in trophoblasts enhances the cytotoxicity of natural killer cells and impairs trophoblast invasion resulting in poor placentation and pregnancy loss [8,47]. Beclin-1 is essential in regulating the initiation of the auto-phagosome process and is often used for the detection of autophagy activity. A previous study confirmed the activation of autophagy by Beclin-1 in first trimester and term control placentae [20]. This is in line with the present study, where we found expression of Beclin-1 both in first and term control placentae. Importantly, Beclin-1 expression was significantly reduced in EMC both on mRNA and protein level, underlining the notion that physiological autophagy levels are important for the sustainability of normal pregnancy. 

In healthy first trimester pregnancies, low oxygen induces hypoxia inducible factors-1α (HIF-1α), thereby initiating the expression of pro-apoptotic proteins that can disrupt the inhibitory interaction of Bcl-2 (and Bcl-extra-large; Bcl-xL) with Beclin-1 to activate autophagy [49]. Interestingly, we found lower levels of Bcl-2 in EMC patients as compared to first trimester and term controls. From a mechanistic point of view, the current literature suggests that autophagy inhibition is due to nitrosylation, which inactivates JNK1 and IKKβ signaling pathways, thus reducing Bcl-2 and AMPK phosphorylation [50]. In this context, our findings suggest that suppression of autophagy could stimulate apoptosis in trophoblasts by activation of pro-apoptotic Bcl-2-associated X protein (Bax). Furthermore, caspase activation during apoptosis can contribute to digest several proteins that are essential for the autophagic machinery to operate, leading to cell demise in EMC [51,52,53]. Previous findings suggested that induction of autophagy by hypoxia supports trophoblast invasion [54], while other studies associated increased autophagy with a reduction in trophoblast invasion due to the inhibition of HIF-1α [55]. Our data showed significantly reduced HIF-1α expression in first trimester as compared to term controls (Supplementary data, Figure S1) and a tendency towards higher HIF-1α levels in EMC placental tissues compared to term controls. These results further support the notion of increased hypoxia in EMC, which is accompanied by downregulated autophagy pathways.

Various studies have shown that OS, which accompanies excessive and unbalanced ROS production, is related to adverse effects on pregnancy and fetal development [56]. In this study, we comprehensively investigated mechanisms that have been implicated in both placentation and imbalance between OS and antioxidant defenses. In this context, we studied not only proteins involved in autophagy processes (Beclin-1, Bcl-2, LC3, and p62) and hypoxia (HIF-1α) but also OS-related processes, which could contribute to destroy the integrity of the cells [12,57]. OS was determined by evaluating TOS, TAC, and antioxidant defenses by quantifying catalase activity and total GSH. It is evident that autophagy in the placenta is activated by OS to protect the cells from apoptosis; therefore, impairment of autophagy leads to OS accumulation [9,15]. Moreover, Bcl-2 modulates ROS signaling, protects the metabolic OS-induced cell death [58], and is reported to have direct or indirect antioxidant effects [59,60,61]. Since we found autophagy inhibition significantly diminished Bcl-2 expression in EMC, we examined the level of OS by measuring TAC and TOS in maternal serum and placentae from EMC and controls. Concurrent with the increase in TOS, there was a significant decrease in TAC, both in maternal serum and EMC placental tissues, as reported previously only in maternal serum [62]. In line with this, we found similar trends for the OS-related enzyme catalase and for total GSH concentration in placental tissues and maternal serum. Our results indicate a highly significant decrease in catalase activity and GSH content in EMC maternal serum and placental tissues compared to controls. Catalases and GSH are known to breach the peroxidation chain reactions by trapping the oxygen free radicals, maintaining redox balance and intracellular homeostasis, and thus, preventing depletion of cellular thiols/protein thiols and lipid peroxidation [63,64]. Excessive production and accumulation of ROS cause damage to cellular organelles, as well as to cellular and extracellular macromolecules such as nuclear and mitochondrial DNA, lipids, and protein destroying the integrity of the cells [65]. Our data further demonstrate increased DNA oxidative damage (represented by 8-OHdG levels), elevated lipid peroxidation products (represented by elevated MDA levels), and enhanced protein oxidation damage (represented by carbonyl contents) both in EMC placental tissues and maternal serum. These observations are in agreement with previous documented studies [62,66,67]. Excessive DNA damage to placenta cells caused by OS, and hence, increased DNA damage markers were also reported in other pregnancy complications [68,69]. Evidently, our comprehensive analyses of OS by measuring TOS, TAC, cellular damage markers, and enzymatic activity revealed increased oxidative damage to macromolecules including DNA, lipids, and proteins as well as reduced antioxidative potential in EMC. However, the reduced antioxidant capacity may not reflect that the antioxidant defense system per se is defective, but could be a consequence of the increased OS. Thus, it could be speculated that due to the severely increased OS, overconsumption of antioxidants occurs, which reflects in an apparently reduced antioxidant capacity. This is supported by data demonstrating that healthy pregnancies in the first trimester have higher levels of antioxidants than women with EMC [19,70].

ABC transporters have diverse physiological functions with different cellular and subcellular localizations from the plasma membrane to intracellular compartments. Since the placenta is the exchange barrier between the maternal and fetal circulation, maternal lipid levels and cholesterol trafficking via placental ABC cholesterol transporters have been suggested [71]. Several members of ABC transporters have been found in the endo-lysosomal system, which consists of endosomes, autophagosomes, and lysosomes [35], and thus, their aberrant expression can be accompanied by defects in the autophagy pathway. Therefore, we hypothesized that dysregulated autophagy modulates the expression of selected placental ABC transporters (ABCA1, ABCG1, and ABCG2) in response to OS, and the abnormal expression of these placental membrane transporters may contribute to placental dysfunction in EMC. Differential ABCA1 expression in the placenta has been reported in primary antiphospholipid syndrome [72], preeclampsia [73], and spontaneous preterm deliveries [74]. Knockdown of ABCA1 in mice contributed to severe embryonic arrest, neonatal death, and fetal loss [75]. In our study, we did not find significant changes in ABCA1 expression in EMC. However, our results showed significant reduction in ABCG1 expression in EMC both on the transcript and protein level. Involvement of ABCG1 in maternal-to-fetal cholesterol transport was confirmed in a recent study on maternal supraphysiological hypercholesterolemia [76], and its decreased expression was associated with decreased efflux [24]. Thus, the diminished expression of ABCG1 may contribute to reduced export of cholesterol to the growing fetus in EMC, and such a reduction in maternal–fetal cholesterol transport during early in pregnancy could compromise fetal development [77]. Beside adequate maternal–fetal cholesterol transport by placental transporters, maternal cholesterol levels are important for the growing fetus in early pregnancy [78] as low concentration of maternal LDL, HDL, and total cholesterol levels were associated with adverse pregnancy outcomes such as intrauterine growth restriction [79,80]. In this context, we detected significantly lower maternal serum lipid levels in the first trimester compared to term, but no differences between EMC and gestational-age-matched controls. These results corroborate previously reported findings that highest maternal cholesterol synthesis in liver is usually in the third trimester, although it already starts during the first trimester [81].

In addition to cholesterol efflux, ABCA1 and ABCG1 also prevent placental accumulation of cytotoxic oxidized cholesterol metabolites known as oxysterols in conditions of OS [24]. Therefore, both ABCA1 and ABCG1 are important to protect against toxic effects of oxysterols on placental and fetal development and placental function, thereby reducing the risks associated with pregnancy complications such as preeclampsia [30,82]. Our results showed a significant downregulation of ABCG1 expression in EMC in combination with higher OS levels. This may result in an accumulation of oxysterols, which negatively affects placentation and leads to oxysterol-induced apoptosis affecting trophoblasts [24] and endothelial cells in the placenta [26].

Importantly, ABCG2, another ABC transporter with the highest expression in the uterus and placenta [31,83], was found to be differentially regulated in EMC. ABCG2 plays an important protective role for maintaining normal physiological function and protecting the cells during OS through efflux of toxic substances, drugs, and a variety of carcinogens [84]. In normal placenta, chemical inhibition of ABCG2 or knockdown of ABCG2 expression significantly increased the sensitivity of placental trophoblasts to apoptotic injury in response to certain stress factors [34]. It has been demonstrated that autophagy triggered by various stressors is augmented in the presence of ABCG2, resulting in reduced cell death with increased cell survival [32]. Interestingly, we found increased ABCG2 mRNA expression as well as protein expression in EMC placentae. The upregulation of ABCG2 in EMC placentae could indicate an adaptive and protective response to alleviate OS in the pathophysiological situation of concomitantly reduced autophagy levels. 

**Figure 10:**
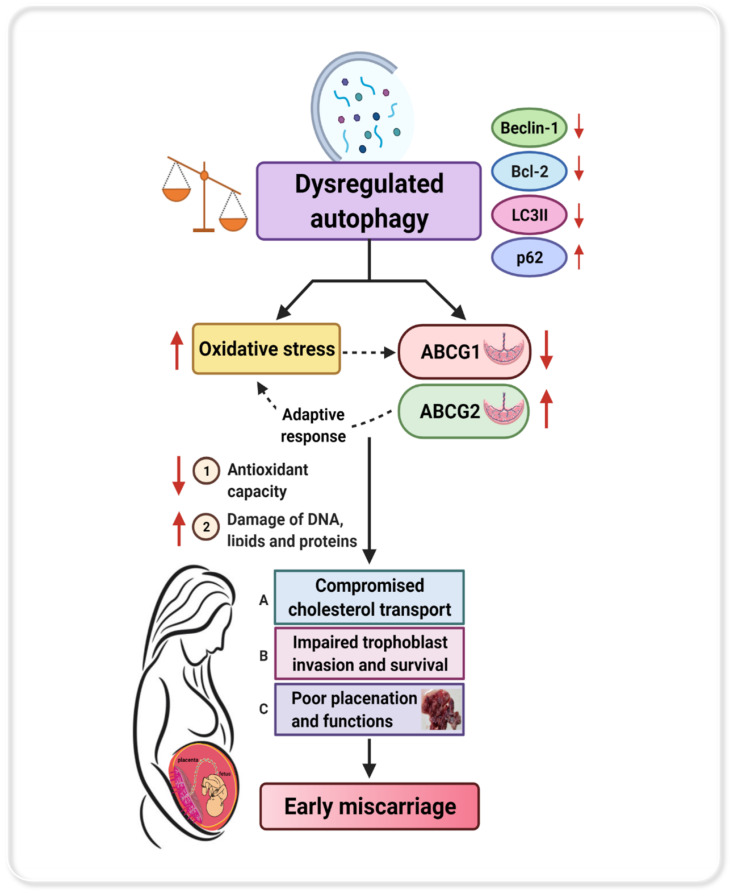
**Schematic representation and proposed working model summarizing the results in EMC patients.** Our findings suggest that a decreased autophagy status triggers Bcl-2-dependent oxidative stress in EMC patients. The decreased expression of ABCG1 is associated with dysregulated autophagy processes. The elevated ABCG2 expression represents a protective feedback mechanism under inhibited autophagic conditions. The aberrant gene expression of placental ABC transporters affects materno-fetal cholesterol transport in EMC. Impaired total antioxidant capacity and increased oxidative stress may further contribute to EMC (created with BioRender).

The main findings of this project as well as a proposed working model are summarized in Figure 10: we demonstrated that autophagy is reduced in EMC patients. Due to the inhibited autophagy status, Bcl-2-dependent OS is triggered leading to the damage of macromolecules. The expression of ABCG1 is downregulated, which contributes to reduced export of cholesterol to the growing fetus in EMC. This pathological situation leads to an increased expression of ABCG2 as a protective response to insults associated with dysregulated autophagy. Taken together, inhibited autophagy processes in combination with increased oxidative toxicity reduce the ability of the placental protection system to mitigate the harmful effects of stress affecting materno-fetal health.

## 5. Conclusions

In conclusion, our findings demonstrate that autophagy is an indispensable process and seems to play an essential role for a successful term pregnancy. Further studies are warranted to determine the underlying mechanisms and regulatory pathways, which are compromised in EMC pregnancies.

## Figures and Tables

**Table 1:** Anthropometric and clinical data of early miscarriage (EMC) patients, 1st trimester, and term controls.

Characteristics	Term Control	First Trimester	EMC
Number of individuals	19	20	20
Delivery mode/type	Cesarean section (17) Vaginal delivery (2)		Incomplete (EMC)
Gestational age (weeks)	37.52 ± 2.1	10.11 ± 1.9 **‡**	10.15 ± 1.9 **‡**
Parity	2.68 ± 1.77	1.00 ± 0.79 **†**	1.35 ± 1.73 **†**
Maternal age (years)	28.8 ± 4.0 (23–35)	28.3 ± 4.8 (20–35)	28.0 ± 4.999 (20–35)
Height (cm)	163.42 ± 1.81	164.54 ± 1.24	163.86 ± 2.41
Weight pregravid (kg)	65.11 ± 7.5	62.19 ± 5.7	62.78 ± 7.2
BMI pregravid (kg m^−2^)	24.4 ± 2.8	23.0 ± 2.0	23.4 ± 2.7
Weight (kg) peripartum	78.3 ± 6.9	65.6 ± 5.8 **‡**	66.28 ± 7.4 **‡**
BMI (kg m^−2^) peripartum	29.3 ± 2.6	24.2 ± 2 **‡**	24.7 ± 2.7 **‡**

Data are represented as mean ± SD (standard deviation). Differences between the first trimester and the term control group, EMC vs. term control group, EMC vs. first trimester were analyzed by Kruskal–Wallis test followed by Dunn’s multiple comparison test; **‡**: *p* ≤ 0.001, **†**: *p* ≤ 0.01. Gestational age of placentae from the Swiss cohort: 39 ± 0.88 weeks (term control; *n* = 5), 8.80 ± 1.14 weeks (1st trimester; *n* = 6). Abbreviations: BMI, body mass index.

## Data Availability

The data that supports the findings of this study is contained within the article or supplementary material or are available on request from the corresponding authors.

## Supplementary Materials

The following supplementary data are available online at https://www.mdpi.com/article/10.3390/antiox10111742/s1: Figure S1: qRT-PCR analysis of hypoxia-inducible factor 1 (HIF-1α) in placental tissues, Table S1: Primer sequences used to quantify autophagy, ABC transporters, and reference genes by RT-qPCR.
