# Supplementary material for: Dysregulated Autophagy Leads to Oxidative Stress and Aberrant Expression of ABC Transporters in Women with Early Miscarriage
Source: Antioxidants (Basel). 2021 Oct 30;10(11):1742. doi: 10.3390/antiox10111742 (PMC8614945; doi:10.3390/antiox10111742)
Supplement: Supplementary file 1 [file antioxidants-10-01742-s001.zip › antioxidants-1390931-supplementary.pdf]

Supplementary data

Manuscript: Dysregulated Autophagy Leads to Oxidative Stress and Aberrant Expression of ABC Transporters in Women with Early Miscarriage

by Saira Shahnawaz et al.

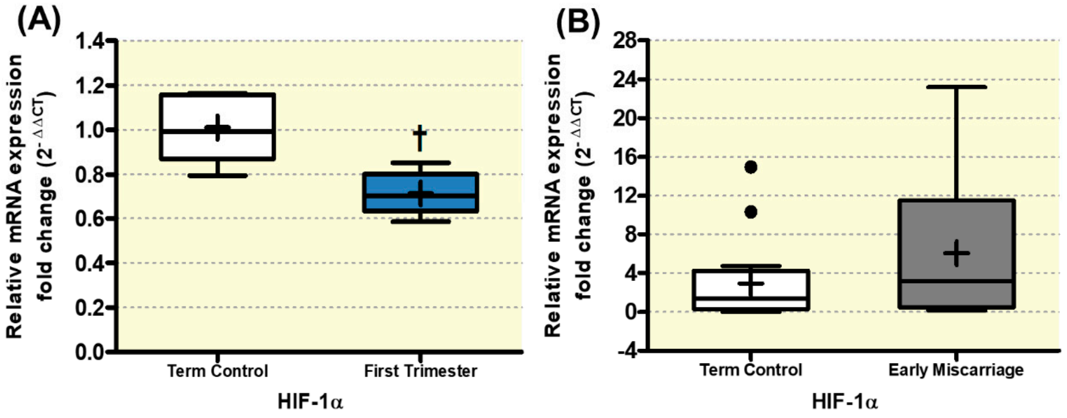

Figure S1. qRT-PCR analysis of hypoxia-inducible factor 1 (HIF-1α) in placental tissues. (A) 1st and 3rd trimester controls (n=6 and n=5, respectively), **(B)** EMC vs term controls (n=20 and n=19, respectively). Data are presented as mean (x), median (-), and Tukey box and whiskers (1.5-times interquartile range). mRNA expression by RT-qPCR are presented as $2^{-\Delta\Delta CT}$ ($\Delta\Delta Ct$ = Ct value of target gene— CT value of the mean of house keepers, i.e B-actin, GAPDH, YWHAZ, B2MG, L19, hTBP, hsUBQ) and normalized to the mean of controls. Significantly reduced HIF-1α expression was observed in 1st trimester as compared to term controls. Significant differences were evaluated by using two-tailed Mann-Whitney test; 1st vs 3rd trimester (A) and EMC vs term controls (B): † ($P \leq .01$).

Table S1. Primer sequences used to quantify autophagy, ABC transporters and reference genes by RT-qPCR

Gene	Forward sequence, 5'-3	Reverse sequence, 5'-3'	Gene Accession Number	fragment length [bp]
		Autophagy		
BECN1	CCCGTGGAATGGAATGAGATTA	CCGTAAGGAACAAGTCGGTATC	NM_003766.5	113
Bcl-2	GCGACTCCTGATTCATTG	CTTCCTCTGTGATGTTGTATT	NM_000657.3	157
LC3-I	TCCCGGACCATGTCAACAT	ACCATGCTGTGCTGGTTCAC	NM_181509.3	106
LC3-II	ACCATGCCGTCGGAGAAG	ATCGTTCTATTATCACCGGGATTTT	NM_001085481.3	115
P62	AGGCGCACTACCGCGAT	CGTCACTGGAAAAGGCAACC	NM_003900.5	51
		ABC Transporters		
ABCA1	CCACATTTTTGCCTGGGACG	AGCGATTCTCCCCAAACCTT	NM_005502.4	88
ABCG1	AACATGGAGGCCACTGAGAC	GGCCACCAACTCACCACTAT	NM_207629.2	228
ABCG2	GAGGCAAATCTTCGTTAT	CCATCACAACATCATCTT	NM_001348989.2	144
		Hypoxia		
HIF-1α	TCCATGTGACCATGAGGAAA	CCAAGCAGGTCATAGGTGGT	NM_001243084.2	251
		Reference Genes		
β-actin	AACTCCATCATGAAGTGTGACG	GATCCACATCTGCTGGAAGG	NM_001101.5	234
GAPDH	GCTCCTCCTGTTCGACAGTCA	ACCTTCCCCATGGTGTCTGA	NM_001357943.2	80
YWHAZ	CCGTTACTTGGCTGAGGTTG	AGTTAAGGGCCAGACCCAGT	NM_001135699.2	143
B2MG	GATGAGTATGCCTGCCGTGTG	CAATCCAAATGCGGCATCT	NM_004048.4	114
L19	CCAACTCCCGTCAGCAGATC	CAAGGTGTTTTTCCGGCATC	NM_000981.4	101
TBP	GTTCTGGGAAAATGGTGTGC	GCTGGAAAACCCAACTTCTG	NM_001172085.2	100
UBC	TCGCAGCCGGGATTTG	GCATTGTCAAGTGACGATCACA	NM_021009.7	64

Gene names: *BECN1*, Beclin-1; *Bcl-2*, B-cell lymphoma 2; *LC3-I*, Microtubule-associated protein light chain 3-I; *LC3-II*, LC3-phosphatidylethanolamine conjugate; *P62*, SQSTM1 gene (Sequestosome-1); *ABCA1*, ATP Binding Cassette Subfamily A Member 1; *ABCG1*, ATP-binding Cassette Subfamily G Member 1; *ABCG2*, ATP-binding cassette super-family G member 2 (BCRP, breast cancer resistance protein); *HIF-1α*, hypoxia-inducible factor 1; *β-actin*, Beta-actin; *GAPDH*, Glyceraldehyde-3-phosphate dehydrogenase; *YWHAZ*, 14-3-3 protein zeta/delta; *B2MG*, beta-2 microglobulin; *L19* (RPL19), ribosomal protein L19; *TBP*, TATA-box-binding protein; *UBC*, ubiquitin.
